# Supplementary material for: Preparation, Identification and Preliminary Application of the Fenvalerate Monoclonal Antibody in Six Kinds of Dark Tea
Source: Foods. 2023 Mar 3;12(5):1091. doi: 10.3390/foods12051091 (PMC10001202; doi:10.3390/foods12051091)
Supplement: Supplementary file 1 [file foods-12-01091-s001.zip › foods-2178805-supplementary.pdf]

Supplementary Materials

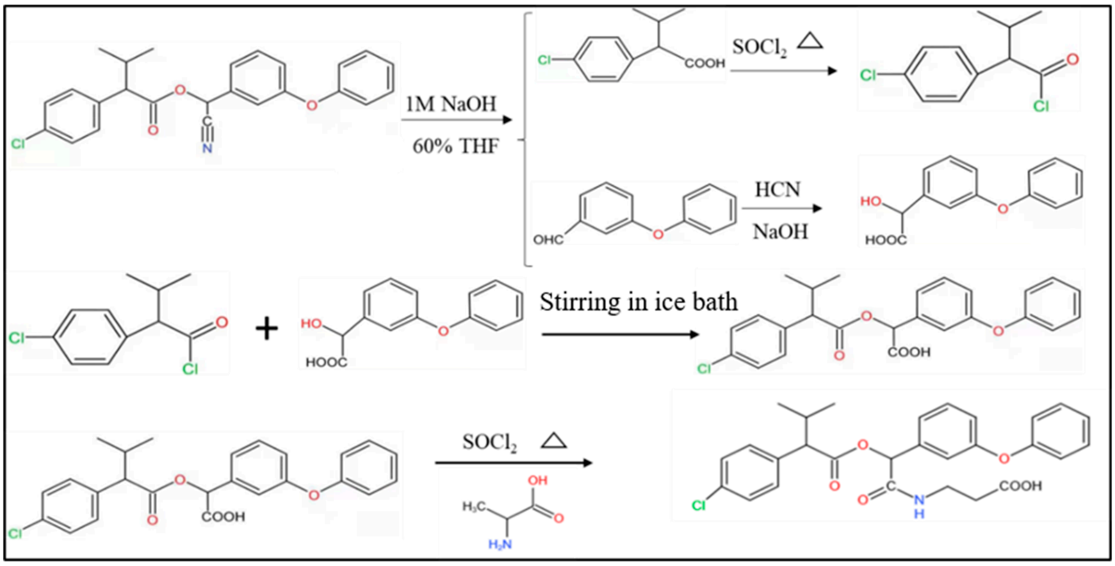

Figure S1. The synthetic procedure of Fen hapten.

Table S1. Titer determination of fenvalerate monoclonal antibody (McAb).

Antibody	Dilution ratio of McAb							
number	3000	9000	27000	81000	243000	729000	2187000	negative
1B6	3.912	3.845	3.73	3.45	1.633	1.269	0.554	0.175
2A11	3.971	3.946	3.757	3.342	2.245	1.046	0.551	0.202
5G2	3.938	3.918	3.58	3.086	2.021	0.987	0.334	0.167
